# An Insight into the Excitation States of Small Molecular Semiconductor Y6

**DOI:** 10.3390/molecules25184118

**Published:** 2020-09-09

**Authors:** Xianshao Zou, Guanzhao Wen, Rong Hu, Geng Dong, Chengyun Zhang, Wei Zhang, Hao Huang, Wei Dang

**Affiliations:** 1 School of Physics and Materials Science, Guangzhou University, Guangzhou 510006, China; xianshao.zou@chemphys.lu.se (X.Z.); gzhwen@e.gzhu.edu.cn (G.W.); chyzhang@gzhu.edu.cn (C.Z.); 2 Division of Chemical Physics, Lund University, 22100 Lund, Sweden; 3 National Research Base of Co-Innovation Center for Micro/Nano Optoelectronic Materials and Devices, Chongqing University of Arts and Sciences, Chongqing 402160, China; hurong_82@cqwu.edu.cn; 4 Department of Biochemistry and Molecular Biology, Shantou University Medical College, Shantou 515041, China; gdong@stu.edu.cn; 5 Medical Informatics Research Center, Shantou University Medical College, Shantou 515041, China; 6 Hebei Key Lab of Optic-Electronic Information and Materials, College of Physics Science and Technology, Hebei University, Baoding 071002, China; hbu_huanghao@163.com

**Keywords:** Y6, organic solar cells, excited state, photoinduced luminescence

## Abstract

Y6 is a new type of non-fullerene acceptor, which has led to power conversion efficiencies of single-junction polymer solar cells over 17% when combined with a careful choice of polymeric donors. However, the excited state characteristics of Y6, which is closely correlated with its opto-electronic applications, are not clear yet. In this work, we studied the excited state properties of the Y6 solution and Y6 film, by using steady-state and time-resolved spectroscopies as well as time-dependent density functional theory (TD-DFT) calculations. UV-Vis absorption and fluorescence simulation, natural transition orbitals (NTOs) and hole-electron distribution analysis of Y6 solution were performed for understanding the excitation properties of Y6 by using TD-DFT calculations. The lifetimes of the lowest singlet excited state in Y6 solution and film were estimated to be 0.98 and 0.8 ns, respectively. Combining the exciton lifetime and photoluminescence (PL) quantum yield, the intrinsic radiative decay lifetimes of Y6 in the solution and film were estimated, which were 1.3 and 10.5 ns for the Y6 solution and film, respectively. Long exciton lifetime (~0.8 ns) and intrinsic radiative decay lifetime (~10.5 ns) of Y6 film enable Y6 to be a good acceptor material for the application of polymer solar cells.

## 1. Introduction

Over recent decades, the polymer solar cell (PSC) has attracted tremendous attention, due to the advantages of low cost, light weight and the applications in flexible devices [1,2,3,4,5]. The power conversion efficiency (PCE) of PSCs increases rapidly due to the recent development of material design and device optimization [6,7]. Currently, the bulk heterojunction structured PSCs, based on conjugated polymer donors and non-fullerene acceptors have achieved a PCE exceeding 18% [8,9], showing a bright future for practical applications.

**Figure 1:**
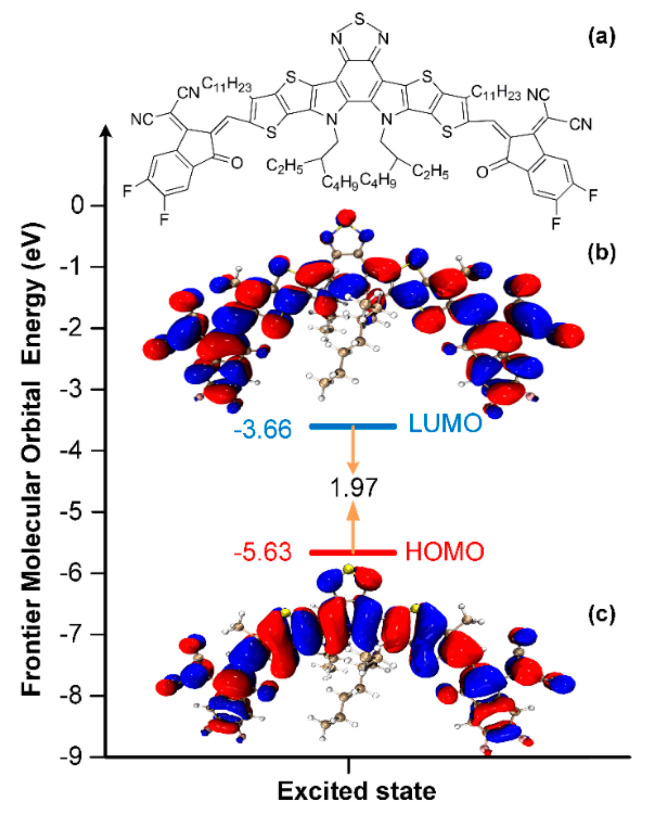
(**a**) Molecular structure and electron density contours of (**b**) LUMO level and (**c**) HOMO level for Y6 with dielectric constant of 4.7113 (Chloroform solution) by TD-DFT with B3LYP/Def2-TZVPP. The isosurface value was set at 0.01 a.u.

**Figure 2:**
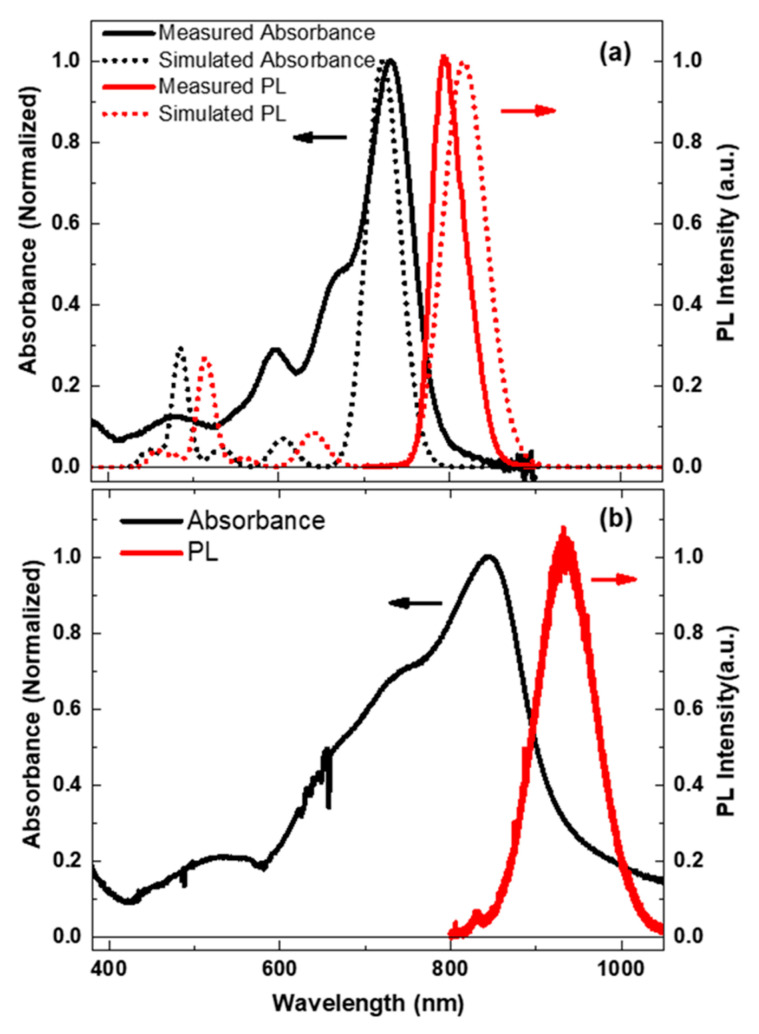
(**a**) Experimental measured (solid lines) and simulated (dot lines) absorption and fluorescence spectrum of Y6 in chloroform solution; the simulations were conducted by TD-DFT with B3LYP/6-31G*, and the vibration contribution was not included in the calculation. A Gaussian function with a full width at half-maximum (FWHM) of 0.11 eV was employed for the simulated spectrum. (**b**) Experimental measured (solid lines) absorption and fluorescence spectrum of Y6 film. The excitation wavelength of experimental measured fluorescence of Y6 solution and Y6 film was 600 nm and 700 nm, respectively.

Recent advances of low-bandgap non-fullerene acceptors significantly improve the absorption of the active layer in the near-infrared region, and thus rapid the increase of PCE. Very recently, the application of a new star of non-fullerene acceptor Y6 (also known as BTP-4F, as shown in Figure 1a), (2,2′-((2*Z*,2′*Z*)-((12,13-bis(2-ethylhexyl)-3,9-diundecyl-12,13-dihydro[1,2,5]thiadiazolo[3,4-e]thieno[2″,3′:4′,5′]thieno[2′,3′:4,5]pyrrolo[3,2-g]thieno[2′,3′:4,5]thieno[3,2-b]indole-2,10-diyl)bis (methanylylidene))bis(5,6-difluoro-3-oxo-2,3-dihydro-1Hindene-2,1-diylidene))dimalononitrile), has successfully promoted the PCE of PSCs over 15% [10,11,12,13,14,15,16,17,18]. To the best of our knowledge, most of the reported PSCs with PCE > 16% contain Y6 or Y6 derivatives [12,13,14,15,16,17,18]. In general, a high-performance PSC requires high absorption efficiency, high charge photogeneration efficiency, low charge recombination, and small energy offset to minimize voltage losses [19,20,21]. Besides the absorption of the active layer, charge photogeneration and recombination processes are other crucial factors for high-performance PSC [22,23]. Since charge photogeneration and recombination processes in Y6 based solar cells, such as exciton diffusion and dissociation, are closely correlated with the excited state of Y6, understanding the excited state properties is essential for its applications. However, the excited state properties of Y6, such as radiative and nonradiative decay processes, are not clear yet. In this work, the excited state properties of Y6 solution and Y6 film were studied by using steady-state and time-resolved spectroscopies as well as time-dependent density functional theory (TD-DFT) calculations. UV-Vis absorption and fluorescence simulation, natural transition orbitals (NTOs), and hole-electron distribution analysis of Y6 solution were performed for understanding the excitation properties of Y6 by using TD-DFT calculations. Combining the simulations of UV-Vis absorption and fluorescence, the peaks in the absorption and photoluminescence spectrum are assigned. From time-resolved photoluminescence (TRPL) measurements, we found that the lifetimes of the lowest excited state in Y6 solution and film were 0.98 and 0.8 ns, respectively. Combining the exciton lifetime and PL quantum yield, the intrinsic radiative decay lifetime of Y6 in solution and film were estimated, which were 1.3 and 10.5 ns for the Y6 solution and film, respectively. The long exciton lifetime and intrinsic radiative decay lifetime of Y6 film enable it to be a good acceptor material for the application of PSCs.

## 2. Results and Discussion

### 2.1. Molecular Structure and Steady-State Optical Properties

Figure 1a shows the chemical structure of Y6 molecular which demonstrates the acceptor-donor-acceptor (A-D-A) structure employing an electron-deficient core-based central fused ring with a benzothiadiazole core. The acceptor of Y6 consists of two planar units, with a twist in the center. TD-DFT calculations were firstly carried out to understand the electronic structure as well as excited state properties of Y6 with B3LYP/6-31G* and B3LYP/Def2-TZVPP basis sets by Gaussian 16 revision B.01 [24,25,26,27]; the detailed calculation methods can be found in supporting information S1. The most stable structure of Y6 is shown as the two largest side chains directed toward the central core. The electron density of the lowest unoccupied molecular orbital (LUMO) and the highest occupied molecular orbital (HOMO) of excited state are presented in Figure 1b,c, Figures S1 and S5. It is shown that the electronic cloud distribution of HOMO tended to delocalize over both the donor and acceptor units, while for LUMO the electron density was mainly located on the acceptor units, indicating that the acceptor units have the strong electron-accepting ability. By applying TD-DFT calculation at B3LYP/Def2-TZVPP level of theory, the HOMO energy level of Y6 was calculated as −5.63 eV, which is close to the experimental value of −5.65 eV by cyclic voltammetry [10], while the LUMO energy level was calculated as −3.66 eV, which slightly deviates from the experimental value of −4.1 eV [10].

Figure 2a shows the absorption and emission spectra of Y6 dilute solution. The absorption spectrum of Y6 shows a peak at ~730 nm and two shoulders at 660 nm and 590 nm, respectively. The ~730 nm peak is due to excitation from the ground state to the lowest excited state. Natural transition orbitals (NTOs) analysis by TD-DFT with B3LYP/Def2-TZVPP shows the transition of S_1_ corresponding to π→π*, and the maximum contribution from the NTO pair was 98.69%. The shoulders at 660 and 590 nm may originate from the excitation to higher electronic or vibrational levels. In comparison to the absorption spectrum, we observed a pronounced Stokes shift of the PL spectrum, which is due to energy losses between excitation and emission. Typically, the excitation could not change the geometry of nuclear much, and we would expect a similar spacing of the vibrational levels between the excited states and the ground state [28]. In this case, the PL spectrum is typically a mirror image of the absorption spectrum when the PL is from the lowest excitation state to the vibrational levels of the ground state. However, Figure 2a shows the breakdown of mirror symmetry between absorption and emission, and the emission spectra of Y6 appeared structureless compared to the absorption spectrum. 

To determine 660 and 590 nm peaks of the absorption spectrum origin from higher vibrational levels of the lowest excited state or the higher electronic states, simulations of the absorption and emission spectrum of Y6 with a dielectric constant of 4.7113 (Chloroform solution) were performed by TD-DFT with the B3LYP/6-31G* level of theory, as shown in the dotted lines in Figure 2a. By comparing the simulated and experimental measured absorption, we can see that the peaks of 730 and 590 nm fit the lowest two electronic transitions (739 and 618 nm peaks) in the simulated spectrum. Thus, 730 and 590 nm peaks in the absorption spectrum of the Y6 solution can be attributed to the transition from the ground state to the lowest two electronic transitions, while the 660 nm peak origin from the transition of the ground state to the 0-1 vibration band of the lowest singlet electronical state (S_1_). We note that the 660 nm peak was absent in the simulation; this is because the vibration of the excited state was not included in the simulation. The 730 and 590 nm absorption peaks can be characterized by HOMO (H)→LUMO (L) (98.5%) and H→L + 1 (96.5%), respectively. The oscillator strength *f* for the 730 nm peak was found to be the highest among the 10 lowest calculated excitations. The overlap function between the hole and electron distribution of the lowest two transitions (S_0_→S_1_, S_0_→S_2_) was more than 60%, and both transitions were local excitation (LE) characters. For the convenience of readers, positions and oscillator strength, the excited-state analysis-based values, natural transition orbital pairs [29], the overlap of electron-hole isosurface density maps [30] of the 10 lowest-energy electronic transitions, and selected electron density contours of selected molecular orbitals were calculated by a multifunctional wavefunction analyzer (Multiwfn) version 3.7 [31] and shown in Supporting information (S2:TD-DFT with B3LYP/Def2-TZVPP, S3: TD-DFT with B3LYP/6-31G*, Figures S1–S10, Tables S1–S5). We found that the experimental measured PL was similar to the simulated fluoresce from S_1_, suggesting the experimental observed PL origin from the lowest exited state S_1_.

As known in Figure 2, the absorption maximum of Y6 was red-shifted approximately 90 nm in the thin film casts from chloroform, which could be induced by the aggregation of the molecular backbone in the solid state [32]. Moreover, both absorption and emission peaks in films were broadened, in comparison to that in the solution, suggesting that the energy state distribution was broadened because of intermolecular interactions in the Y6 film. We note the absorption of Y6 film covered a broad wavelength range of 600–930 nm, which could compensate the absorption spectra of the reported most of the polymer donors in the near IR region.

### 2.2. Excited State Properties of Y6 Solution and Film

**Figure 3:**
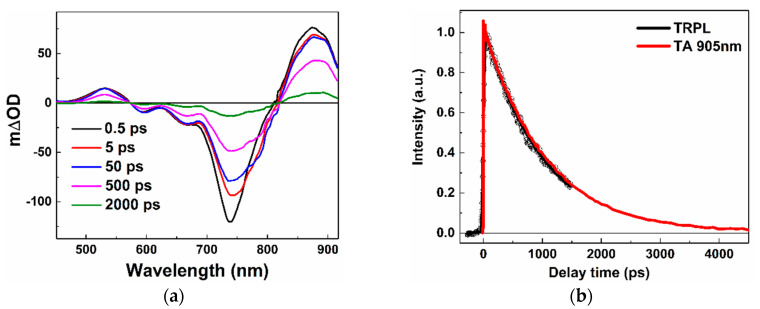
(**a**) Transient absorption spectra of Y6 solution at indicated delay times after photoexcitation at 700 nm with an excitation fluency of 2 × 10^15^ photons cm^−2^. (**b**) Comparison of time-resolved photoluminescence (TRPL) and transient absorption (TA) kinetics in the Y6 solution.

In order to understand excited state decay processes of Y6, we carried out femtosecond time-resolved absorption spectroscopy measurements in the visible-near IR region. Figure 3a depicts the representative transient spectra of Y6 in the chloroform solution. The spectra show the excited-state absorption peaked at ~530 and ~875 nm; the bleaching of the ground state absorption at ~590, ~660, and ~730nm; and the stimulated emission from 730 to 800 nm. To assign the excited-state absorption signal around 900 nm, we compared TRPL and transient absorption kinetics at 900 nm (as shown in Figure 3b), we found that both kinetics exhibited single exponential decay behavior with a lifetime of ~980 ps. Hence, the absorption peak around 900 nm in transient absorption and the photoluminescence signal origin from the same excited state (S_1_). In the very recent study, Zhang et al. found that the primary of the excitation was local excitation (LE) state in the Y6 solution [33].

In the TRPL measurement, PL kinetics was determined by intrinsic radiative decay and nonradiative decay channels. To understand the intrinsic lifetime of the excited state, we analyzed the radiative decay rate based on Einstein coefficients. The Einstein coefficient for stimulated absorption *B*_lu_ that moves from a lower state *l* to an upper state *u* can be expressed as [34,35]

(1) $$B_{lu}=\frac{c\ln10}{hvN_{A}}\int \varepsilon dv$$

where *h* is the Planck constant, *c* is the light speed in vacuum, *ν* is the frequency of the transition, *N*_A_ is Avogadro’s number, and *ε* is the molar extinction coefficient. The spontaneous emission coefficient, *A*_ul_, can be expressed as [35,36]

(2) $$A_{ul}=\frac{8\pi hv^{3}}{c^{3}}B_{lu}$$

Here, *A_ul_* equals the radiation decay rate of the excited state (*k*_r_) in the absence of nonradiative decay channels. The spontaneous emission coefficient (*k*_r_) (8.33 × 10⁸ s^−1^) of Y6 in chloroform solution can be derived from expression (1) and (2). The PL quantum yield (*Φ*) of the excited state of Y6 in chloroform solution can be defined as [28].

(3) $$\Phi =\frac{k_{r}}{k}$$

where *k_r_* and *k* are the radiative and total decay rates of the excited state, respectively. By using the radiative recombination rate estimated from Einstein coefficients and PL decay rate from the TRPL measurement, we obtained a PL quantum yield of ~80%. This parameter was close to the absolute PL quantum yield (~70%) measured via the integration sphere method.

**Figure 4:**
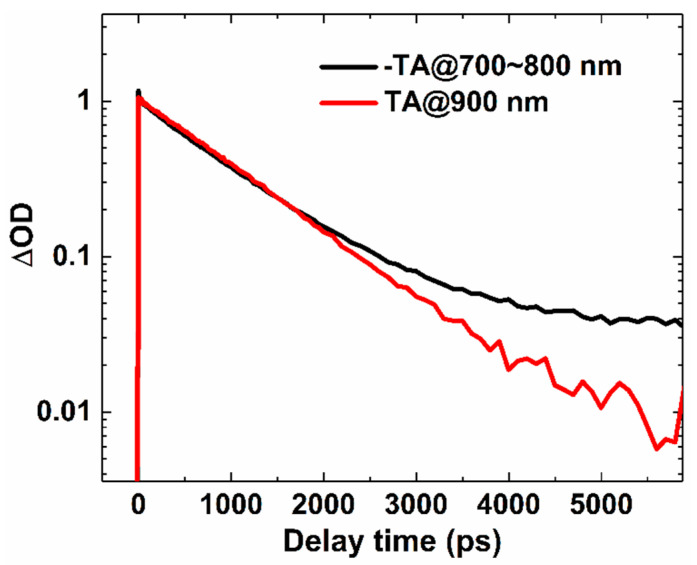
TA kinetics of Y6 in chloroform solution at the indicated probe wavelength after photoexcitation at 700 nm with an excitation fluency of 2 × 10^15^ photons cm^−2^.

To examine whether there were long-lived species besides the S_1_ species, we compared the S_1_ kinetics at 900 nm and the bleaching kinetics in the range of 700–800 nm in TA, as shown in Figure 4. We found that there was long-lived decay beside the S_1_ decay. In semiconductors, many species, such as triplet states and charge states, could induce long-lived signal for the bleaching signal. Y6 is a small molecule, the primary excitation is LE states [33]; accordingly, the long-lived component could not be attributed to the decay of charge states. Considering the formation of triplet states via intersystem crossing is a common process in organic semiconductors, and the decay of triplet states is relatively long, we deduced that the long-lived component observed in the bleaching kinetics of Y6 solution was most likely due to the formation of the triplet state. Further study of this long-lived component is needed.

**Figure 5:**
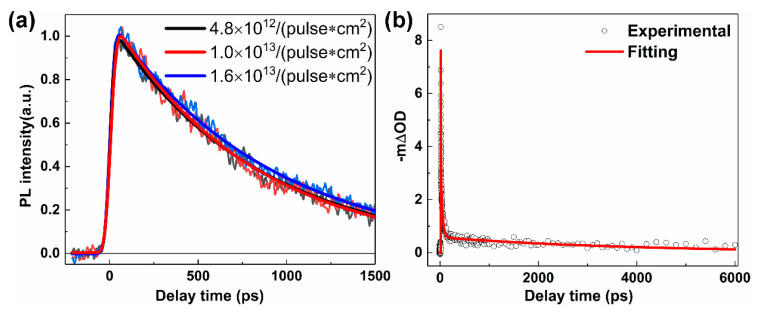
(**a**) TRPL kinetics of Y6 film at indicated excitation fluence after photoexcitation at 775 nm. (**b**) TA kinetics of Y6 film at a probe wavelength of 870 nm after photoexcitation at 700 nm; the excitation was 2 × 10^15^ photons cm^−2^. The solid lines after fitting curves using exponential decay functions; the fitting parameters can be found in supporting information (Tables S6 and S7).

Furthermore, we examined TRPL kinetics of Y6 film after photoexcitation at 775 nm at various excitation fluences, as shown in Figure 5. We found that the kinetics exhibited similar single exponential decay behavior with a PL lifetime of ~800 ps, which was slightly faster than PL decay in solution (~980 ps). Considering the absolute PL quantum yield of Y6 film (~7.5%), we deduced the intrinsic radiative PL lifetime of the excitons in the film, which was ~10.5 ns. We noted that the intrinsic radiative PL lifetime of excitons in the film was much slower than that in the Y6 solution (~1.3 ns). This increased intrinsic exciton lifetime in the film could be correlated with the formation of intermolecular excitons, because of the aggregation of the molecular backbone in the film. Very recently, Zhang et al. found that the intramoiety excited states, which were similar to pseudo charge transfer state or intermolecular exciton, could be formed from local excitation on a time scale of ~0.2 ps in the Y6 film [33]. By comparing TA kinetics of Y6 in solution and film under the same excitation fluence. We found that there was a faster decay process at the early delay time of the Y6 film, which was not shown in the solution. Since the fast decay processes were not shown in TRPL at a relatively low excitation fluency, we deduced that this fast decay process was induced by the exciton annihilation of Y6 at a high excitation power in the TA measurements. Moreover, we noted that the slowest component of TA was ~4 ns, which was much slower than PL lifetime (~800 ps). Considering the strong excitation annihilation processes of Y6 film in TA measurements, the slowest component could be correlated with the charge pairs that formed due to exciton annihilation.

In polymer solar cells, exciton dissociation efficiency is one of the key steps in determining charge photogeneration efficiency of devices, since the exciton dissociation efficiency is determined by ($1-\tau_{\mathrm{blend}}/\tau_{\mathrm{pure}}$), where $\tau_{\mathrm{blend}}$ and $\tau_{\mathrm{pure}}$ are the PL lifetime of the blend film and neat Y6 film [37]. Apparently, a longer lifetime of Y6 exciton ($\tau_{\mathrm{pure}}$) would increase the exciton dissociation efficiency of Y6 in the blend film. We note that the exciton lifetime of Y6 film (0.8 ns) is longer than many classical small molecular acceptors, such as ITIC (0.524 ns) [38], PDI-(1, 2, 3) (<0.7 ns) [39], IDTTBM (0.097 ns) [40], as well as classical polymer acceptors N2200 (~0.059 ns) [37]. The relatively long exciton lifetime of Y6 film would be helpful for the exciton diffusion and dissociation of Y6 in the blend film. A very recent study shows that hole transfer time is very short, and the exciton dissociation efficiency is very high in Y6 based PSC [33,41,42]. Moreover, DFT calculations and experimental studies of Y6 single crystals show that they have high ambipolar charge transport properties [41,43]. It can be seen from the above discussions that Y6 is a very good acceptor material in the application of polymer solar cells.

## 3. Materials and Methods

### 3.1. Sample Information

Y6 was purchased from Solarmer Materials Inc. (Beijing, China) without further processing, and its purity was greater than 99%.

### 3.2. Steady State Absorption and Photoluminescence (PL) Spectrum Measurement

For the absorption measurement, Y6 was first weighed by an electronic balance (ME104/02, Mettler Toledo Inc., Greifensee, Switzerland), and then was dissolved in chloroform (Sigma-Aldrich, St. Louis, MO, USA) to obtain a Y6 solution with a concentration of ~10^−5^ mol/L. The prepared Y6 solution was put into a 1 mm cuvette and measured by a UV-visible spectrometer (UV-2550, Shimadzu, Kyoto, Japan) at room temperature.

Y6 film was prepared by fast solvent evaporation from the prepared chloroform solution on a quartz substrate in air. Before preparation of this film, the quartz substrate was cleaned by ultrasonication in detergent and washed with deionized water, acetone, isopropyl alcohol, and ethanol. The absorption spectrum of Y6 film was measured by the UV-visible spectrometers of Agilent 8453.

The PL spectra of the Y6 solution and film were measured by a standard spectrofluorometer of Fluorolog-3, HORIBA. The excitation wavelength of Y6 solution and film was 600 and 700 nm, respectively. The measurements were conducted at room temperature in the air.

### 3.3. Absolute Photoluminescence Quantum Yield (PLQY) and Time-Resolved Photoluminescence (TRPL) Measurements

PLQY and TRPL were measured in the setups described in [44,45] at Chemical Physics, Lund University. The PL quantum yield system includes continuous wavelength (CW) laser sources, a spectrometer (AvaSpec-ULS2048-USB2-UA-50), and an integrating sphere (HORIBA, Quanta-φ, F3029), which have been specially designed to measure the absolute PLQY of solution and solid samples. The output of the signal from the integration sphere was collected by two 1 inch quartz lenses of 50 mm focal length and focused on the input slit of the spectrometer (AvaSpec-ULS2048-USB2-UA-50). Suitable glass filters were applied to avoid the overexposure of the spectrometer at the excitation wavelength. The measured spectrum was calibrated by using a reference light source (Ocean Optics, Dunedin, FL, USA, LS-1-CAL), and thus, the influence factors such as the reflection index of the integrating sphere, the sensitivity of spectrometer, and the collection efficiency of optical paths can be corrected. For PLQY and TRPL measurements of the Y6 solution, Y6 was dissolved in chloroform (Sigma-Aldrich, anhydrous, ≥99%, contains 0.5–1.0% ethanol as a stabilizer), the freshly prepared solution was put into a 1 cm cuvette, and the cuvette was sealed by parafilm during PLQY and TRPL measurements. For PLQY measurement, the cuvette with Y6 solution was put onto a mount at the center of the integration sphere, and excited by a 660 nm CW laser with an excitation fluency of ~5 mW/cm^2^. For PLQY measurement of the Y6 film, the sample was put at the mount at the bottom of the integrating sphere, and was excited by a 780 nm CW laser with an excitation fluency of ~5 mW/cm^2^. TRPL was measured in a setup described in [44,46]. A Ti:sapphire fs laser (Spectra-Physics, Mountain View, CA, USA, Tsunami, 81 MHz) at 775 nm was used as an excitation source with a pulse duration of 100 fs. The incident angle of the excitation beam was close to the Brewster angle (~70°). PL was collected by two plano-convex lenses with a 50 mm focal length and was focused on the input-slit of the spectrometer (Chromex). The output PL from the spectrograph was directed into the streak camera (Hamamatsu C6860) with a slit width of 30 μm. The measured TRPL image background correction was performed first, after that, shading and spectral sensitivity correction was performed by using a standard calibrating light source (Ocean Optics, LS-1-CAL). The measurements were conducted in the air at room temperature.

### 3.4. Transient Absorption (TA) Measurements

Femtosecond transient absorption measurements were conducted using a Spirtfire ACE 100F (800 nm, pulse width 100 fs, 1 kHz repetition rate, Spectra-Physics) and an ultrafast spectroscopic system (Harpia, Light Conversion) at Hebei University. The output of the fundamental laser was split into pump (95%) and probe (5%) beams. The pump beam was directed through an optical parametric amplification system (TOPAS Prime-F) to generate a 700 nm pump beam, while the probe beam was passed through an optical delay rail and focused onto a Ti:sapphire crystal to generate a white light continuum. The angle between polarization directions of the pump beam and probe beam was set as a magic angle 54.7°, and the optical delay stage provided a probe time window of 8 ns. The fresh prepared Y6 chloroform solution and film were measured at room temperature in the air.

## 4. Conclusions

In this work, the excited state properties of the Y6 semiconductor molecule were studied by using steady-state and time-resolved spectroscopies as well as TD-DFT calculations. Combining the simulation of UV-Vis absorption and fluorescence, the peaks in the absorption and photoluminescence spectrum were assigned. Moreover, PL lifetimes of the lowest excited state in Y6 solution and film were estimated to be 0.98 and 0.8 ns, respectively, via TRPL measurements. Moreover, we found that the intrinsic radiative decay lifetime of Y6 in solution and film can be as long as 1.3 and 10.5 ns for Y6 solution and film, respectively. Long exciton lifetime and intrinsic radiative decay lifetime of Y6 film enable Y6 to be a good acceptor material for the application of polymer solar cells.

## Supplementary Materials

The following are available online. Figure S1: Electron density contours of selected molecular orbitals (TD-DFT with B3LYP/Def2-TZVPP); Figure S2: Simulated UV-vis absorption spectrum and oscillator strength (TD-DFT with B3LYP/Def2-TZVPP); Figure S3: Computed natural transition orbital pairs for S_1_−S_10_ (TD-DFT with B3LYP/Def2-TZVPP); Figure S4: The overlap of electron-hole isosurface density maps between S_0_ and the ten lowest excited energy states (TD-DFT with B3LYP/Def2-TZVPP); Figure S5: Electron density contours of selected molecular orbitals (TD-DFT with B3LYP/6-31G*); Figure S6: Simulated UV-vis absorption spectrum and oscillator strength (TD-DFT with B3LYP/6-31G*); Figure S7: Simulated PL spectrum and oscillator strength (TD-DFT with B3LYP/6-31G*); Figure S8: Computed natural transition orbital pairs for S_1_−S_10_ (TD-DFT with B3LYP/6-31G*); Figure S9: The overlap of electron-hole isosurface density maps between S_0_ and the ten lowest excited energy states (TD-DFT with B3LYP/6-31G*); Figure S10: Presentation of the electron density difference between excited and ground states (TD-DFT with B3LYP/6-31G*); Table S1: Computed positions and oscillator strength of the ten lowest-energy electronic transitions (TD-DFT with B3LYP/Def2-TZVPP); Table S2: Excited-state analysis-based values for the *S*_r_, *D*, *t*, *H*, *E*_coul_, *HDI* and *EDI* of the transitions between S_0_ and the ten lowest excited energy states (TD-DFT with B3LYP/Def2-TZVPP); Table S3: Computed positions and oscillator strength of the ten lowest-energy electronic transitions (TD-DFT with B3LYP/6-31G*); Table S4: Excited-state analysis-based values for the *S*_r_, *D*, *t*, *H*, *E*_coul_, *HDI* and *EDI* of the transitions between S_0_ and the ten lowest excited energy states (TD-DFT with B3LYP/6-31G*); Table S5: Geometrically optimized atomic X/Y/Z coordinates of the Y6 ground state structure; Table S6: Fitting parameters of the TRPL decay shown in Figure 5a; Table S7: Fitting parameters of the TA decay shown in Figure 5b.
