# Supplementary material for: An Insight into the Excitation States of Small Molecular Semiconductor Y6
Source: Molecules. 2020 Sep 9;25(18):4118. doi: 10.3390/molecules25184118 (PMC7570483; doi:10.3390/molecules25184118)
Supplement: Supplementary file 1 [file molecules-25-04118-s001.pdf]

Supporting Information

An Insight into the Excitation States of Small Molecular Semiconductor Y6

Xianshao Zou [1,3], Guanzhao Wen [1], Rong Hu [2], Geng Dong [4,5], Chengyun Zhang [1], Wei Zhang [1,*], Hao Huang [6] and Wei Dang [6,*]

[1] School of Physics and Materials Science, Guangzhou University, Guangzhou 510006, China; xianshao.zou@chemphys.lu.se (X.Z.); gzhwen@e.gzhu.edu.cn (G.W.); chyzhang@gzhu.edu.cn (C.Z.); wzhang@gzhu.edu.cn (W.Z.)

[2] National Research base of Co-innovation Center for Micro/Nano Optoelectronic Materials and Devices, Chongqing University of Arts and Sciences, Chongqing 402160, China; hurong_82@cqwu.edu.cn (R.H.)

[3] Division of Chemical Physics, Lund University, Lund 22100, Sweden

[4] Department of Biochemistry and Molecular Biology, Shantou University Medical College, Shantou 515041, China; gdong@stu.edu.cn (G.D.)

[5] Medical Informatics Research Center, Shantou University Medical College, Shantou 515041, China

[6] Hebei Key Lab of Optic-electronic Information and Materials, College of Physics Science and Technology, Hebei University, Baoding 071002, China; hbu_huanghao@163.com (H.H.); dangwei@hbu.edu.cn (W.D.)

* Correspondence: wzhang@gzhu.edu.cn (W.Z.); dangwei@hbu.edu.cn (W.D.)

Main Contents

1. Computational details.. S1
2. TD-DFT with B3LYP/Def2-TZVPP S2
Table S1. Computed positions and oscillator strength of the ten lowest-energy electronic transitions.. S2
Figure S1. Electron density contours of selected molecular orbitals.. S3
Figure S2. Simulated UV-vis absorption spectrum and oscillator strength S4
Figure S3. Computed natural transition orbital pairs for S_1–S_{10}.. S6
Figure S4. The overlap of electron-hole isosurface density maps between S_0 and the ten lowest excited energy states S7
Table S2. Excited-state analysis-based values for the S_r, D, t, H, E_{coul}, *HDI* and *EDI* of the transitions between the S_0 and ten lowest excited energy states S8
3. TD-DFT with B3LYP/6-31G* S8
Table S3. Computed positions and oscillator strength of the ten lowest-energy electronic transitions.. S8
Figure S5. Electron density contours of selected molecular orbitals.. S9
Figure S6. Simulated UV-vis absorption spectrum and oscillator strength S10
Figure S7. Simulated PL spectrum and oscillator strength.. S10
Figure S8. Computed natural transition orbital pairs for S_1–S_{10}.. S12
Figure S9. The overlap of electron-hole isosurface density maps between S_0 and the ten lowest excited energy states S13
Table S4. Excited-state analysis-based values for the S_r, D, t, H, E_{coul}, *HDI* and *EDI* of the transitions between S_0 and ten lowest excited energy states.. S14
Figure S10. Presentation of the electron density difference between excited and ground states S15
Table S5. Geometrically optimized atomic X/Y/Z coordinates of the ground state structure of Y6 .... S16
4. Fitting parameters in Figure 5.. S19
Table S6. Fitting parameters of the TRPL decay shown in Figure 5a S19
Table S7. Fitting parameters of the TA decay shown in Figure 5b.. S19
References S20

1. Computational details

The original 3-dimensional chemical structure of Y6 was drawn in the open-source molecular editor software package ChemBio3D Ultra 12.0 (Cambridge Inc.). In order to reduce the computational costs, the long alkyl chains of the central unit are replaced by methyl groups. The geometry structure optimization of the ground state of Y6 was performed using density functional theory (DFT) with the hybrid Becke 3-parameter Lee-Yang-Parr (B3LYP) functional [1] and the Pople-style double zeta and one polarization function basis set (6-31G*) [2]. After that time-dependent density functional theory (TD-DFT) calculations were carried out to optimize the geometry of excited state with B3LYP/6-31G*. All the DFT and TD-DFT calculations were carried out employing Gaussian 16 Revision B.01 [3]. Moreover, the single point energy of the ten lowest energy of excited states was calculated by TD-DFT using the polarizable continuum model (PCM) with the dielectric constant of 4.7113 at the level of B3LYP functional with 6-31G* basis set and a balanced basis set of triple zeta valence quality with two sets of polarization function (Def2-TZVPP) [4]. The dominant particle-hole pair contributions and the associated weights were calculated through the national transition orbitals (NTOs) [5] so as to evaluate the properties of electronic transitions from ground state to excited states. Finally, based on the output files of TD-DFT method, multifunctional wavefunction analyzer (Multiwfn) version 3.7 [6] and VMD version 1.9.3 [7] were used to get more transition information such as the theoretical UV-vis absorption and fluorescence spectrum, the electron density of frontier molecular orbitals (FMOs), major molecular orbital (MO) transitions in all excited states, natural transition orbitals (NTOs), the overlap of electron-hole distribution of excited states [8] and the presentation of the electron density difference between excited and ground state.

2. TD-DFT with B3LYP/Def2-TZVPP

Table S1. Computed positions and oscillator strength (*f*) of the 10 lowest-energy electronic transitions of Y6 solution by TD-DFT at B3LYP/Def2-TZVPP level of theory.

State	λ_{cal} (eV, nm)	f^a	Excitation contribution[b] (%)
S_1	1.68 (739)	2.2771	H→L (98.5)
S_2	2.00 (618)	0.1572	H→L+1 (96.5)
S_3	2.25 (539)	0.1024	H→L+2 (96.1)
S_4	2.30 (539)	0.0137	H-1→L (92.6)
S_5	2.35 (527)	0.0471	H→L+3 (79.8), H-2→L (15.0)
S_6	2.48 (500)	0.0916	H-3→L (50.1), H-1→L+1 (43.8)
S_7	2.50 (496)	0.5543	H-2→L (76.6), H→L+3 (15.1)
S_8	2.64 (470)	0.0229	H-1→L+1 (47.9), H-3→L (36.6), H→L+4 (8.8)
S_9	2.66 (467)	0.0127	H→L+4 (74.8), H-2→L+1 (9.5), H-3→L (9.2)
S_{10}	2.74 (453)	0.1106	H-2→L+1 (80.3), H→L+4 (13.6)

[a]Oscillator strengths. [b]H=HOMO, L=LUMO, H-1=HOMO-1, L+1=LUMO+1.

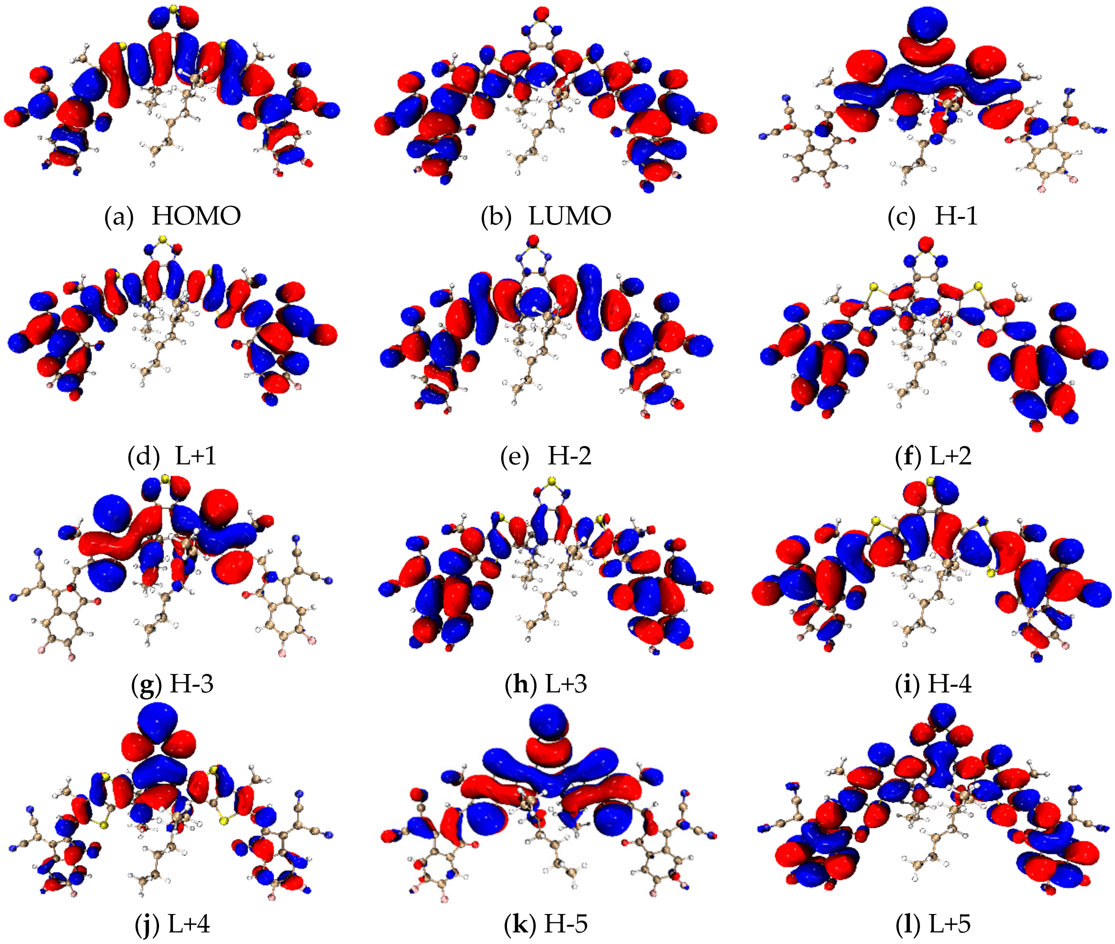

Figure S1. Electron density contours of (a) HOMO, (b) LUMO, (c) HOMO-1, (d) LUMO+1, (e) HOMO-2, (f) LUMO+2, (g) HOMO-3, (h) LUMO+3, (i) HOMO-4, (j) LUMO+4, (k) HOMO-5, (l) LUMO+5 of Y6 solution by TD-DFT at B3LYP/Def2-TZVPP level of theory. The isosurface value was set at 0.01 a.u..

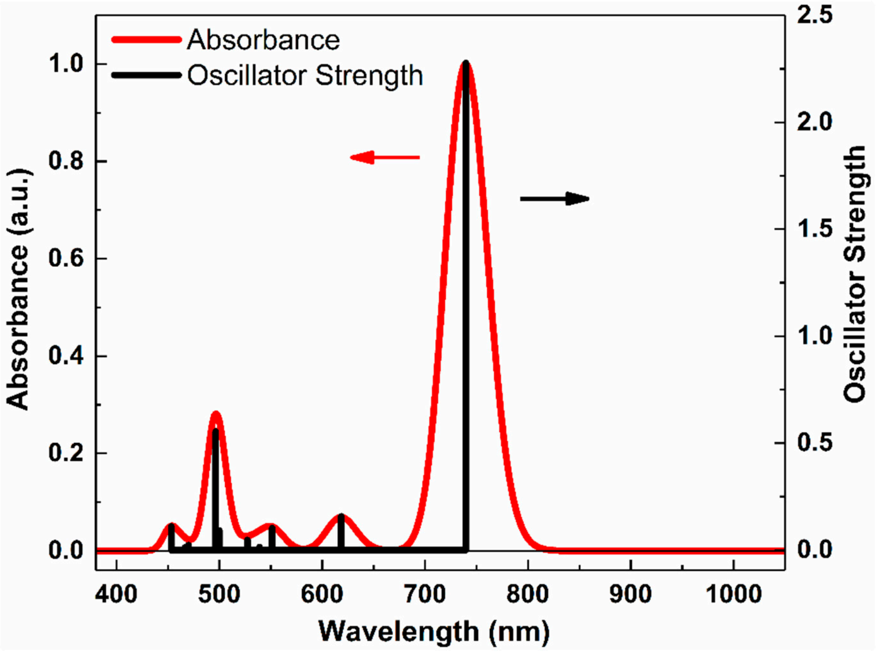

Figure S2. Simulated UV-vis absorption spectrum (red curve) and oscillator strength (black spikes) of the Y6 solution by TD-DFT at B3LYP/Def2-TZVPP level of theory. A Gaussian function with a full width at half-maximum (FWHM) of 0.11 eV was employed.

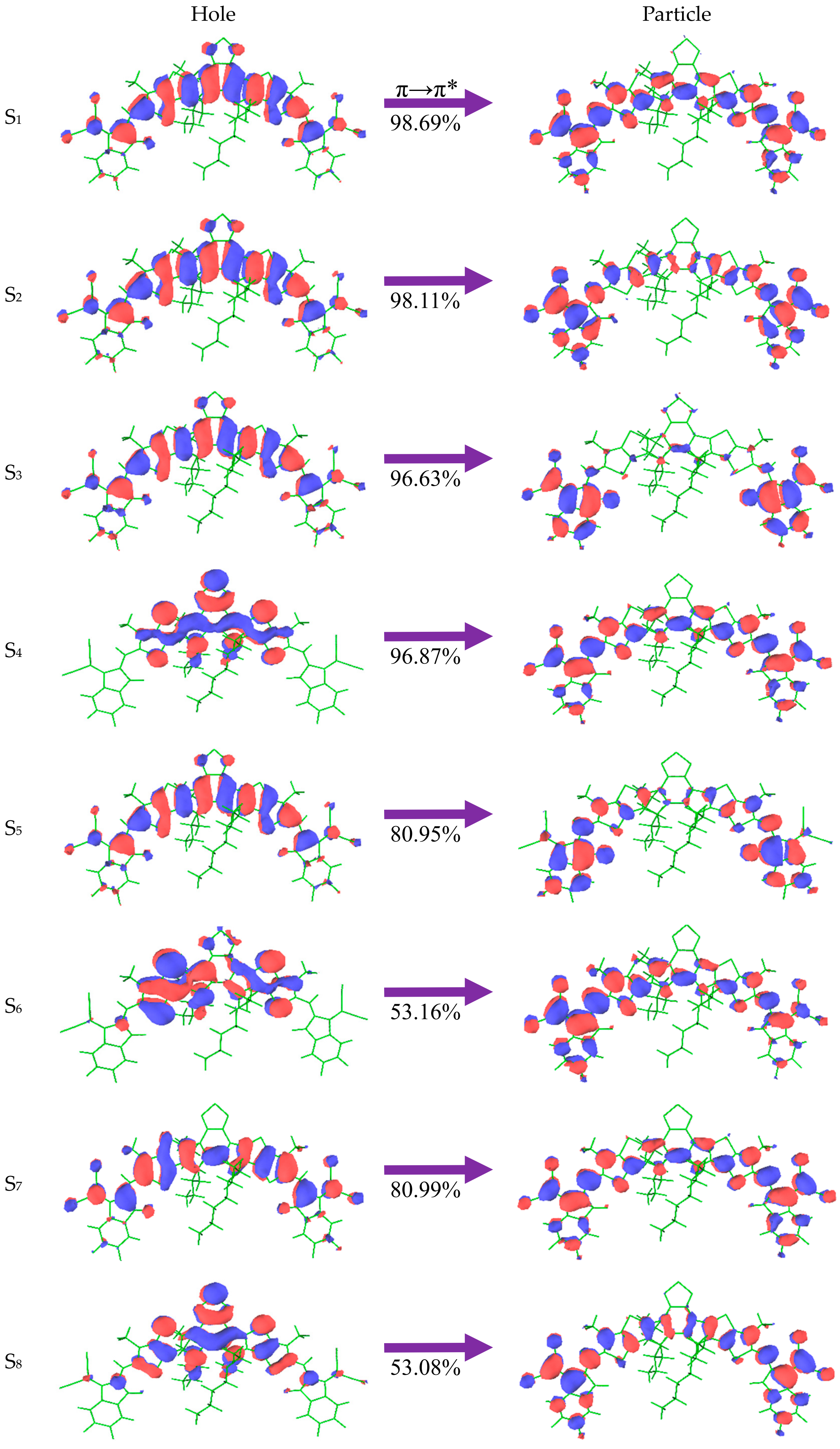
Hole
Particle
S1
π→π*
98.69%
S2
98.11%
S3
96.63%
S4
96.87%
S5
80.95%
S6
53.16%
S7
80.99%
S8
53.08%

S5

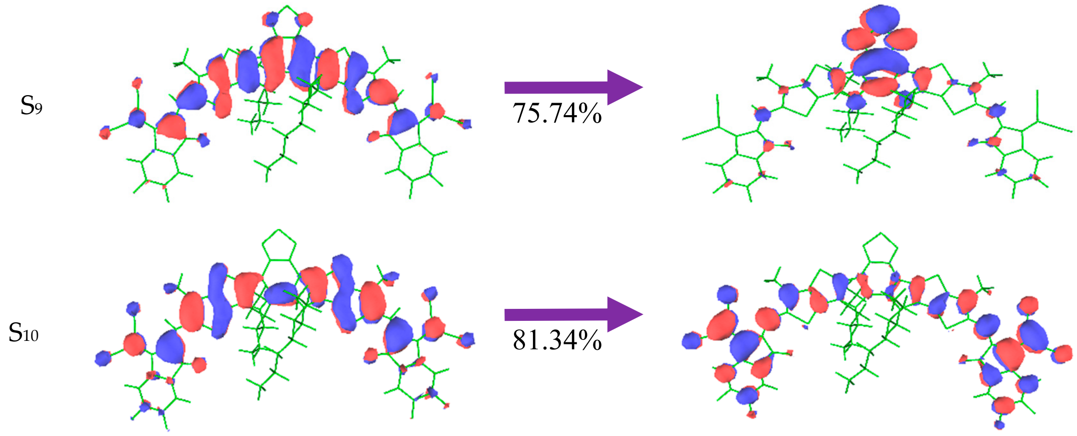

Figure S3. Computed natural transition orbital pairs for S_1–S_{10} of Y6 by TD-DFT at the level of B3LYP/Def2-TZVPP. The isosurface value was set at 0.018 a.u..

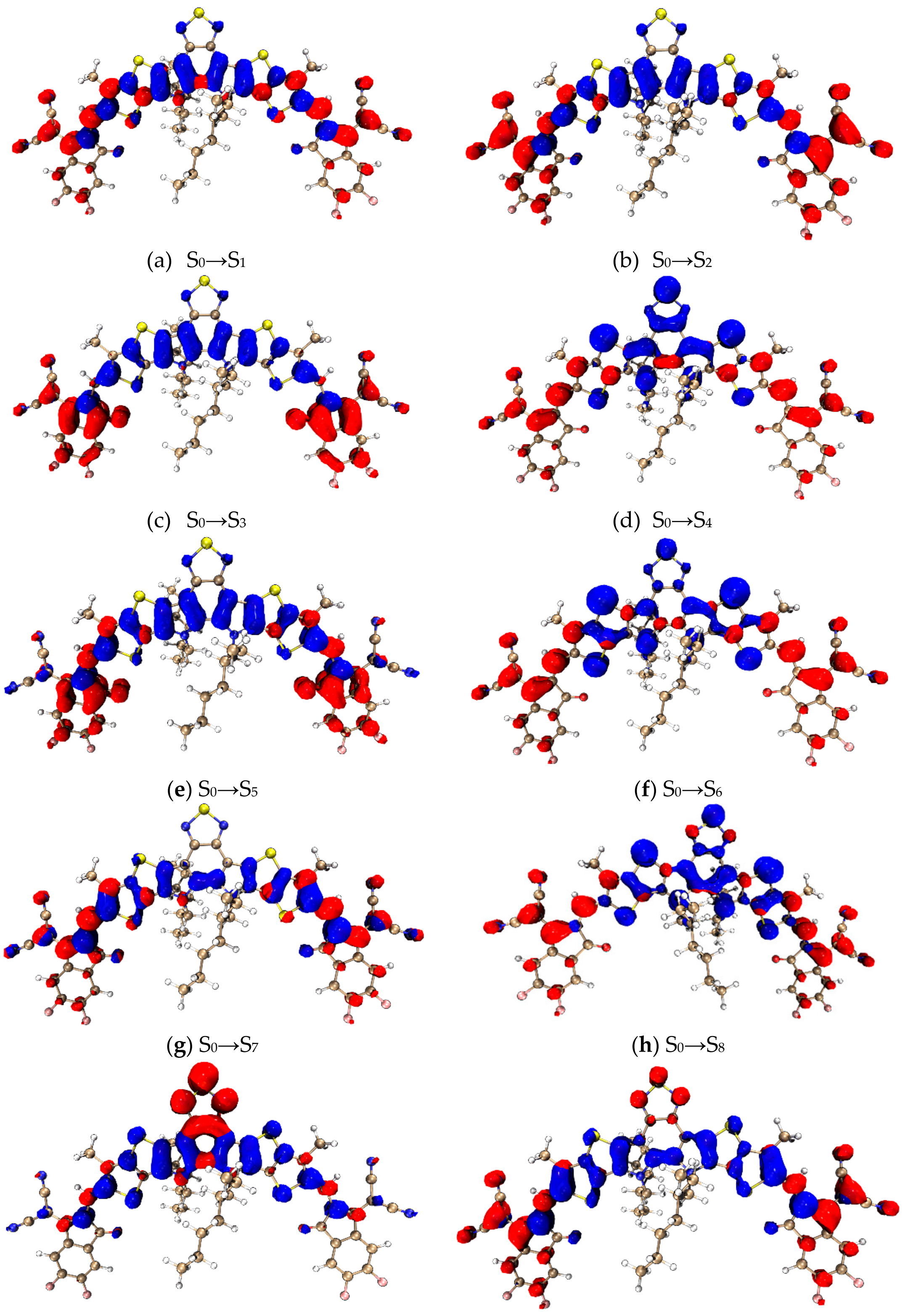

(a) $S_0 \rightarrow S_1$ (b) $S_0 \rightarrow S_2$

(c) $S_0 \rightarrow S_3$ (d) $S_0 \rightarrow S_4$

(**e**) $S_0 \rightarrow S_5$ (**f**) $S_0 \rightarrow S_6$

(**g**) $S_0 \rightarrow S_7$ (**h**) $S_0 \rightarrow S_8$

(**i**) $S_0 \rightarrow S_9$ (**j**) $S_0 \rightarrow S_{10}$

Figure S4. The overlap of electron-hole isosurface density maps between (a) S_0 and S_1, (b) S_0 and S_2, (c) S_0 and S_3, (d) S_0 and S_4, (e) S_0 and S_5, (f) S_0 and S_6, (g) S_0 and S_7, (h) S_0 and S_8, (i) S_0 and S_9, (j) S_0 and S_{10} of Y6 solution by TD-DFT at B3LYP/Def2-TZVPP level of theory. The isosurface value was set at 0.0009 a.u..

Table S2. S_r, D, t, H, E_{coul}, *HDI* and *EDI* of the transitions between S_0 and 10 lowest-energy states in Y6 solution by TD-DFT at the level of B3LYP/Def2-TZVPP.

Transition	D^a (Å)	S_r^b	H^c (Å)	t^d (Å)	E_{coul}^e (eV)	HDI^f	EDI^g	Transition Character
$S_0 \rightarrow S_1$	1.34	0.67	7.50	−2.74	1.94	4.56	4.23	LE
$S_0 \rightarrow S_2$	2.00	0.64	8.02	−2.83	1.82	4.52	4.66	LE
$S_0 \rightarrow S_3$	3.34	0.56	7.72	−0.87	1.75	4.48	5.54	CT
$S_0 \rightarrow S_4$	3.14	0.50	6.31	−0.54	2.07	5.31	4.23	CT
$S_0 \rightarrow S_5$	2.36	0.69	7.59	−2.16	1.88	4.33	4.92	CT
$S_0 \rightarrow S_6$	2.66	0.55	6.72	−1.44	2.02	4.89	4.30	CT
$S_0 \rightarrow S_7$	0.95	0.77	7.99	−3.19	1.84	4.31	4.02	LE
$S_0 \rightarrow S_8$	2.13	0.67	6.84	−1.98	2.04	4.47	4.03	CT
$S_0 \rightarrow S_9$	1.68	0.72	6.09	−2.03	2.35	4.11	6.73	LE
$S_0 \rightarrow S_{10}$	0.68	0.71	8.16	−4.45	1.82	4.14	4.24	LE

[a]the distance between centroid of hole and electron in corresponding directions; [b]the overlap function between hole and electron distribution; [c]the average degree of spatial extension of hole and electron distribution in X/Y/Z direction; [d]the degree of hole and electron in charge transfer direction; [e]exciton binding energy; [f]the hole delocalization index; [g]the electron delocalization index.

3. TD-DFT with B3LYP/6-31G*

Table S3. Computed positions and oscillator strength (*f*) of the 10 lowest-energy electronic transitions of Y6 solution by TD-DFT at B3LYP/6-31G* level of theory.

State	λ_{cal} (eV, nm)	f^a	Excitation contribution[b] (%)
S_1	1.72 (722)	2.2709	H→L (98.7)
S_2	2.05 (605)	0.1601	H→L+1 (96.6)
S_3	2.32 (534)	0.1104	H→L+2 (96.0)
S_4	2.42 (513)	0.0060	H-2→L (69.2), H-1→L (22.8)
S_5	2.42 (513)	0.0141	H→L+3 (65.9), H-1→L (29.5)
S_6	2.55 (485)	0.6055	H-1→L (45.2), H-2→L (26.9), H→L+3 (24.6)
S_7	2.60 (477)	0.0867	H-3→L (45.0), H-1→L+1 (25.7), H-2→L+1 (25.6)
S_8	2.69 (460)	0.0381	H→L+4 (69.1), H-1→L+1 (24.6)
S_9	2.74 (452)	0.0287	H-3→L (42.6), H-1→L+1 (34.5), H→L+4 (17.7)
S_{10}	2.79 (444)	0.0721	H-2→L+1 (65.7), H-1→L+1 (11.3), H→L+4 (9.6)

[a]Oscillator strengths. [b]H=HOMO, L=LUMO, H-1=HOMO-1, L+1=LUMO+1.

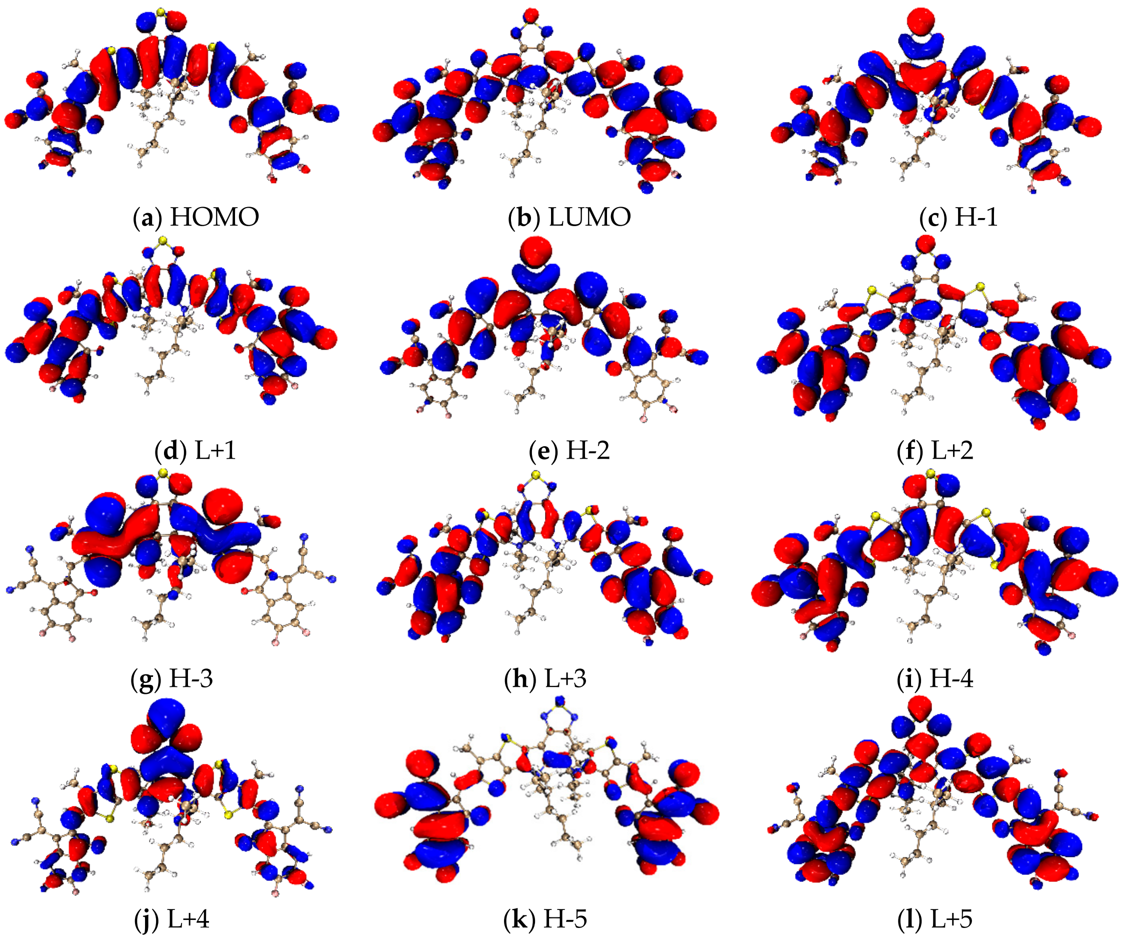

Figure S5. Electron density contours of (a) HOMO, (b) LUMO, (c) HOMO-1, (d) LUMO+1, (e) HOMO-2, (f) LUMO+2, (g) HOMO-3, (h) LUMO+3, (i) HOMO-4, (j) LUMO+4, (k) HOMO-5, (l) LUMO+5 of Y6 solution by TD-DFT at B3LYP/6-31G* level of theory. The isosurface value was set at 0.01 a.u..

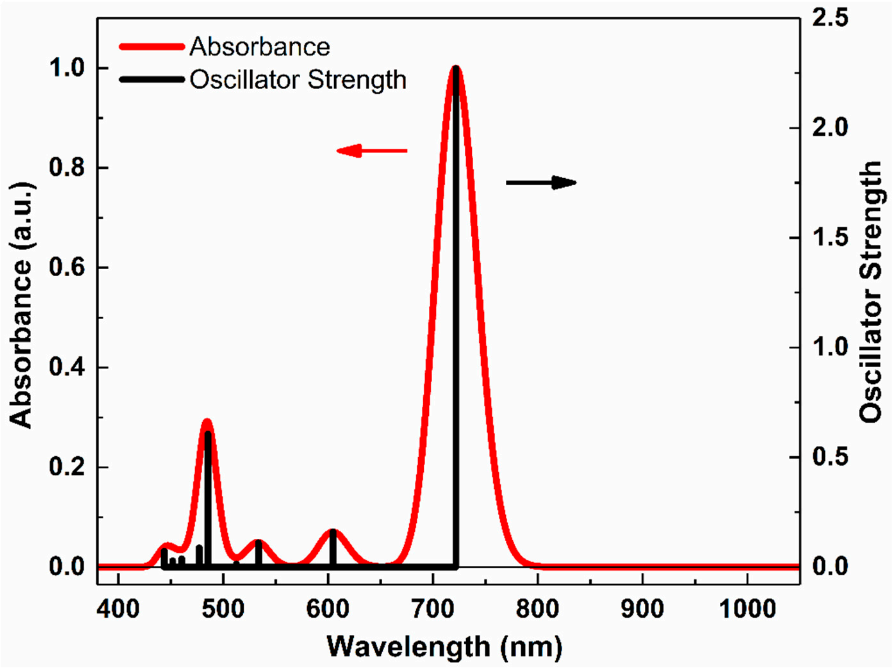

Figure S6. Simulated UV-vis absorption spectrum (red curve) and oscillator strength (black spikes) of the Y6 solution by TD-DFT at B3LYP/6-31G* level of theory. A Gaussian function with a full width at half-maximum (FWHM) of 0.11 eV was employed.

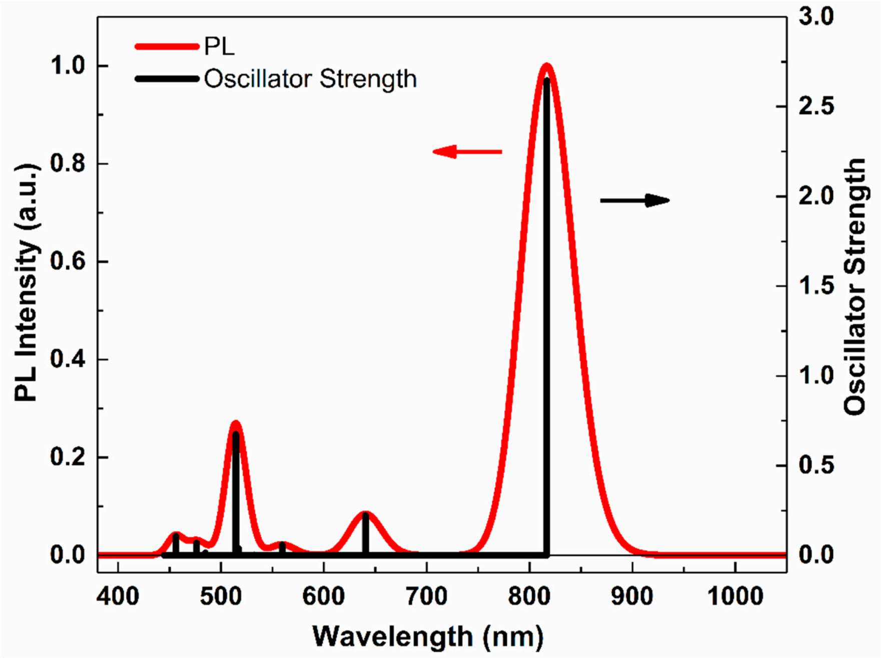

Figure S7. Simulated PL spectrum (red curve) and oscillator strength (black spikes) of the Y6 solution by TD-DFT at level of B3LYP/6-31G*.A Gaussian function with a full width at half-maximum (FWHM) of 0.11 eV was employed.

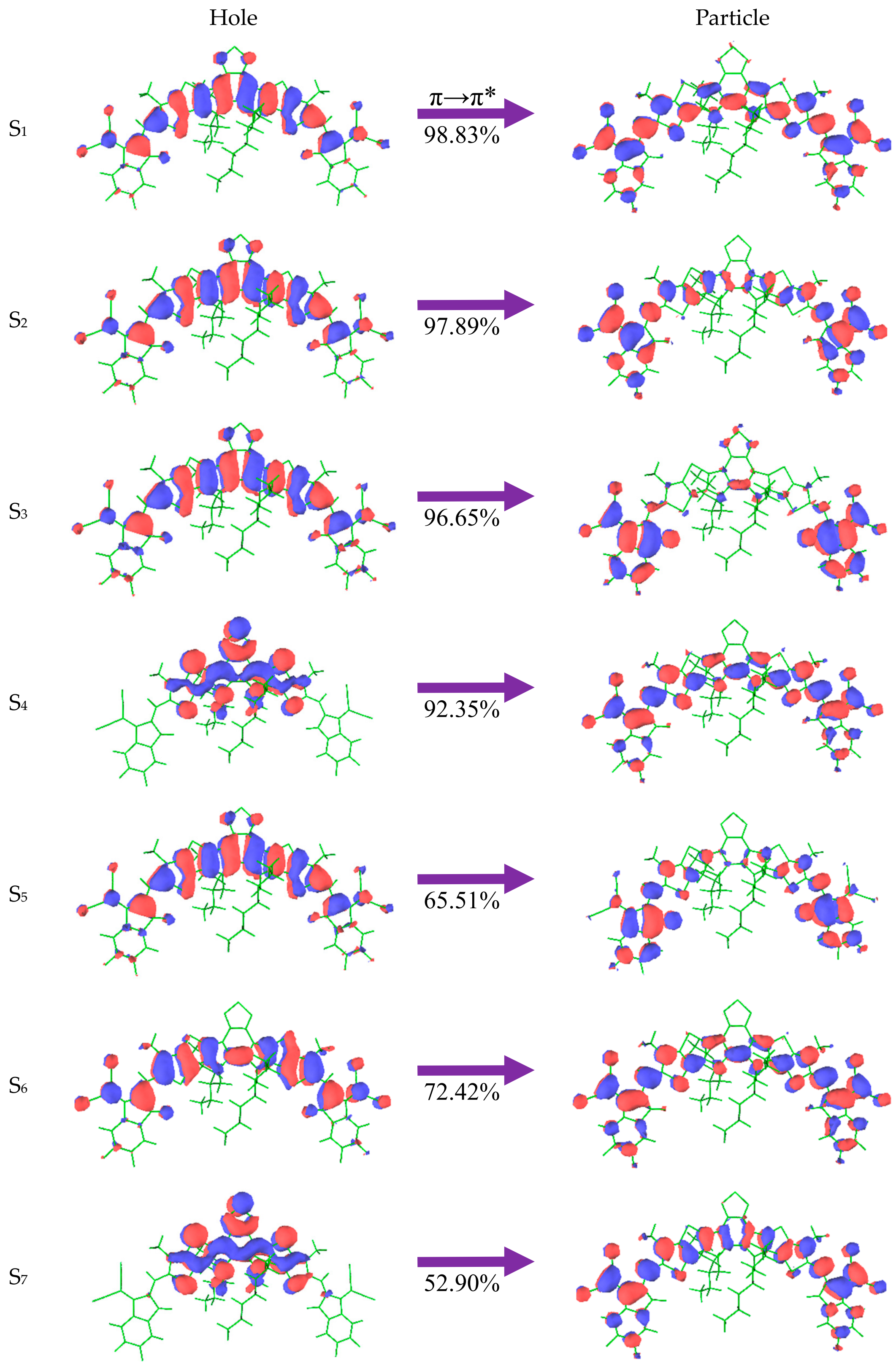

Hole
Particle
S1
π→π*
98.83%
S2
97.89%
S3
96.65%
S4
92.35%
S5
65.51%
S6
72.42%
S7
52.90%

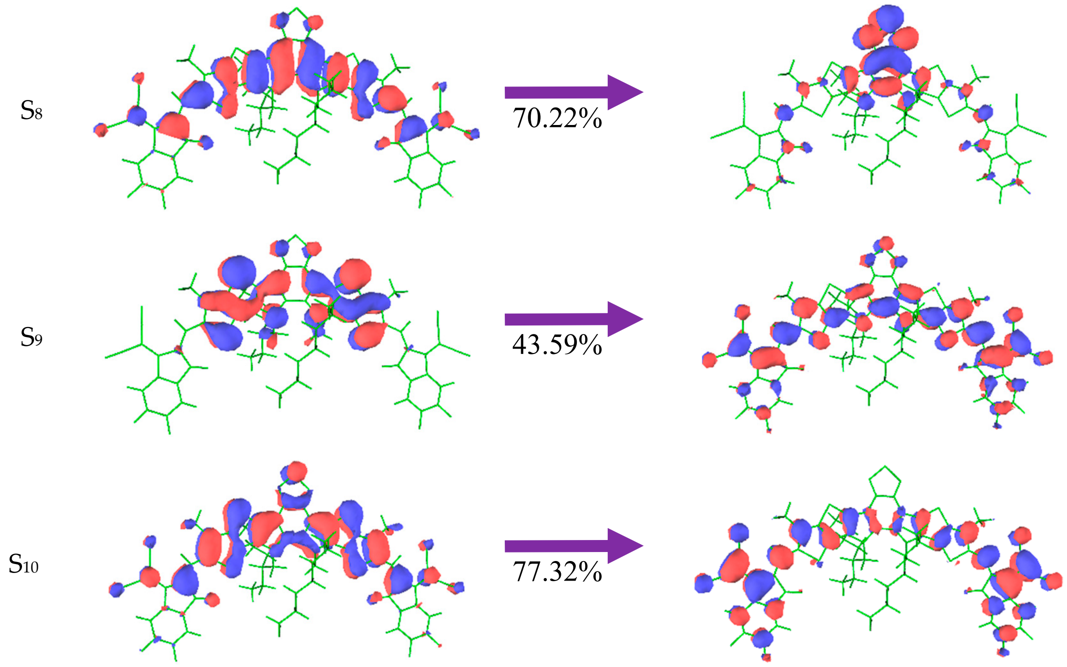

Figure S8. Computed natural transition orbital pairs for S_1–S_{10} of Y6 solution by TD-DFT at B3LYP/6-31G* level of theory. The isosurface value was set at 0.018 a,u,.

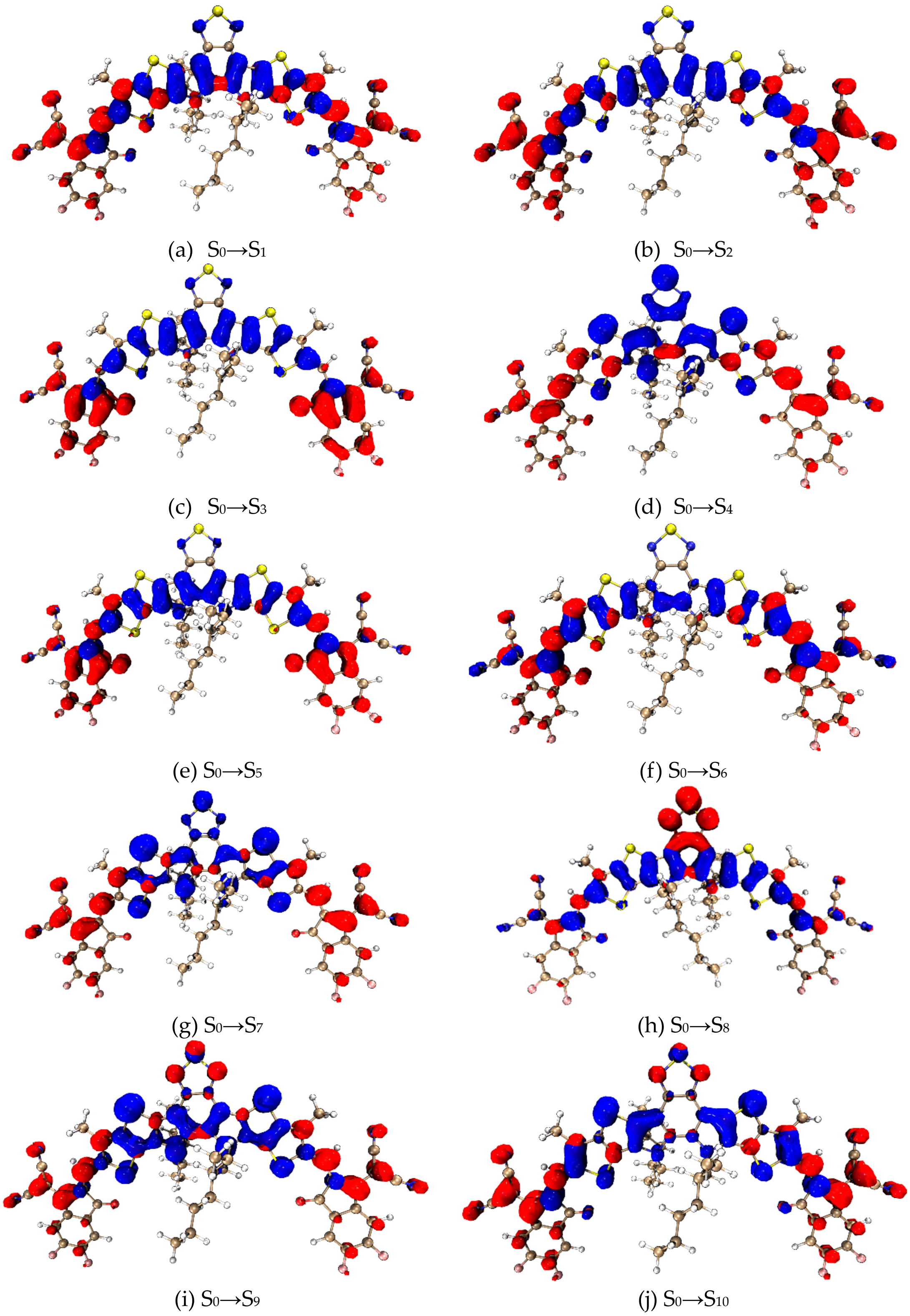

Figure S9. The overlap of electron-hole isosurface density maps between (a) S_0 and S_1, (b) S_0 and S_2, (c) S_0 and S_3, (d) S_0 and S_4, (e) S_0 and S_5, (f) S_0 and S_6, (g) S_0 and S_7, (h) S_0 and S_8, (i) S_0 and S_9, (j) S_0 and S_{10} of Y6 solution by TD-DFT at B3LYP/6-31G* level of theory. The isosurface value was set at 0.0009 a.u..

Table S4. S_r, *D*, *t*, *H*, E_{coul}, *HDI* and *EDI* for the transitions between S_0 and 10 lowest-energy excited states in Y6 solution by TD-DFT at the level of B3LYP/6-31G*.

Transition	D^a (Å)	S_r^b	H^c (Å)	t^d (Å)	E_{coul}^e (eV)	HDI^f	EDI^g	Transition Character
$S_0 \to S_1$	1.29	0.67	7.45	−2.75	1.95	4.64	4.24	LE
$S_0 \to S_2$	1.95	0.64	7.96	−2.84	1.83	4.61	4.67	LE
$S_0 \to S_3$	2.98	0.60	7.80	−1.42	1.81	4.54	5.57	CT
$S_0 \to S_4$	3.09	0.57	6.35	−0.62	2.07	5.12	4.16	CT
$S_0 \to S_5$	2.18	0.71	7.65	−2.56	1.88	4.23	4.58	CT
$S_0 \to S_6$	1.06	0.78	7.92	−3.62	1.86	4.33	4.03	LE
$S_0 \to S_7$	2.63	0.55	6.70	−1.32	2.02	4.95	4.35	CT
$S_0 \to S_8$	1.60	0.71	6.61	−2.36	2.18	4.29	6.33	LE
$S_0 \to S_9$	1.34	0.73	6.87	−2.78	2.07	4.11	3.98	LE
$S_0 \to S_{10}$	1.48	0.72	7.84	−3.21	1.86	3.84	4.21	LE

[a]the distance between centroid of hole and electron in corresponding directions; [b]the overlap function between hole and electron distribution; [c]the average degree of spatial extension of hole and electron distribution in X/Y/Z direction; [d]the degree of hole and electron in charge transfer direction; [e]exciton binding energy; [f]the hole delocalization index; [g]the electron delocalization index.

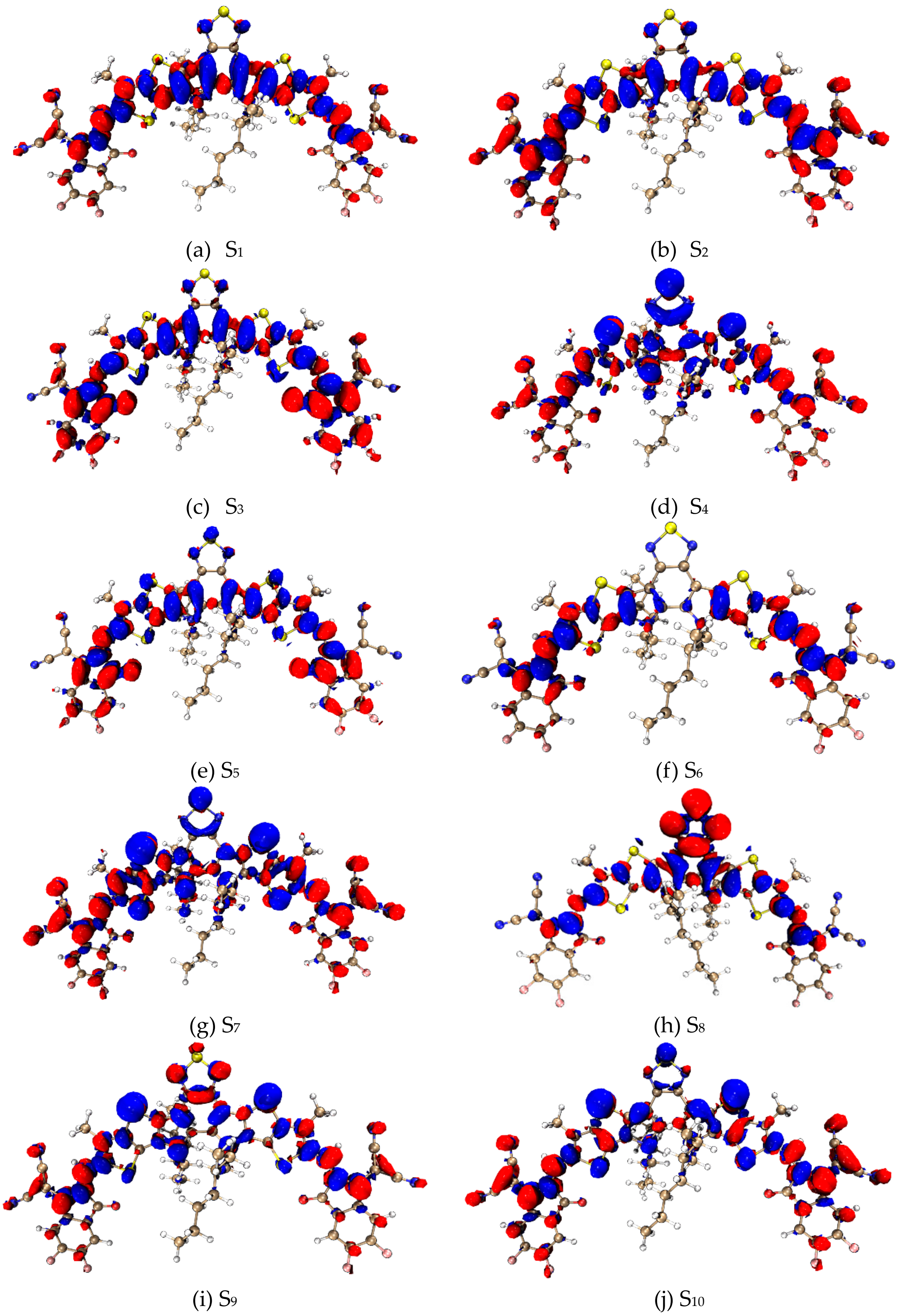

(a) S_1 (b) S_2
(c) S_3 (d) S_4
(e) S_5 (f) S_6
(g) S_7 (h) S_8
(i) S_9 (j) S_{10}

Figure S10. The electron density difference between (a) S_0 and S_1, (b) S_0 and S_2, (c) S_0 and S_3, (d) S_0 and S_4, (e) S_0 and S_5, (f) S_0 and S_6, (g) S_0 and S_7, (h) S_0 and S_8, (i) S_0 and S_9, (j) S_0 and S_{10} of Y6 solution by TD-DFT at B3LYP/6-31G* level of theory. The isosurface value was set at 0.0005 a.u. (blue, electron density decreases upon transition; red, electron density increases upon transition).

Table S5. Geometrically optimized atomic X/Y/Z coordinates of the ground state structure of Y6.

N	3.334	−1.423	−6.840
N	4.17	−2.722	−4.838
C	2.203	0.914	−4.155
C	3.477	−0.987	−3.226
C	3.595	−1.567	−4.530
C	2.879	0.293	−3.054
C	3.115	-0.823	-5.677
C	2.401	0.402	-5.469
C	4.396	1.124	6.548
H	-2.067	1.302	-4.117
H	9.715	2.247	-2.467
C	-4.505	8.431	-10.889
S	4.703	-2.765	-1.212
C	-2.715	6.63	-7.968
N	3.038	0.702	-1.730
S	1.361	1.391	-8.049
O	-2.366	6.578	-5.561
H	-2.804	-0.508	-2.507
F	-5.514	10.828	-4.765
H	6.386	3.927	0.772
H	5.625	-0.389	9.368
C	-5.449	10.348	-7.071
H	-2.363	1.919	1.131
H	5.684	5.541	0.591
H	-1.951	5.41	-9.477
C	0.312	2.776	-7.697
C	0.234	3.082	-6.325
C	5.045	-2.293	1.633
C	-1.943	5.568	-8.405
C	-4.205	8.992	-5.496
C	4.058	-0.643	0.264
F	4.845	1.762	10.546
S	3.982	0.139	1.809
S	0.784	4.453	-6.022
C	5.006	0.123	7.323
N	-2.599	5.775	-11.787
C	3.939	2.322	7.083
C	3.658	-0.345	-1.056
C	-3.773	8.242	-6.583
H	-5.361	9.878	-9.142
C	6.916	2.094	-2.098

H	-0.371	2.499	2.583
C	9.180	1.626	-3.195
C	4.608	2.578	-1.010
C	3.160	2.100	-1.272
H	9.742	1.655	-4.135
C	-5.016	9.596	-8.156
H	5.458	3.562	-2.753
C	-1.119	4.610	-7.77
C	5.168	0.328	8.700
H	2.551	2.218	-0.369
C	6.35	-3.261	6.018
C	-2.872	7.069	-6.565
C	1.682	1.242	-6.339
C	4.513	3.973	-0.350
C	-1.024	3.271	2.159
C	4.643	-1.921	0.346
H	-0.404	4.153	1.954
C	5.996	-2.171	6.870
H	6.908	1.072	-1.692
H	7.243	1.496	-4.15
H	6.505	4.782	-0.776
C	4.948	-0.656	5.078
H	-2.528	-1.171	-4.118
C	1.025	2.200	-5.557
C	4.762	-1.273	2.579
H	-3.892	8.758	-4.484
C	5.37	-1.014	6.435
C	7.741	2.116	-3.391
H	-0.119	4.374	-10.528
S	4.105	-2.851	-6.492
H	6.682	-3.483	2.408
C	5.091	-1.436	3.944
N	1.324	2.002	-4.213
H	-1.526	3.331	-10.375
F	-6.269	11.387	-7.266
C	-2.036	-0.691	-3.265
C	0.367	2.427	-3.166
H	9.199	0.593	-2.827
O	3.861	1.340	4.212
C	-1.719	2.775	0.887
H	-1.320	-1.410	-2.848
C	-1.330	0.596	-3.708
H	0.095	2.628	-10.355

H	3.472	3.079	6.462
F	3.691	3.640	9.038
C	-3.517	7.524	-8.808
C	4.336	0.688	5.136
H	5.814	-4.216	1.0540
C	-3.079	6.539	-11.049
C	5.692	-3.612	1.958
C	4.711	1.526	9.236
C	-3.688	7.496	-10.182
C	5.465	2.560	-2.300
H	0.904	2.927	-2.362
C	-5.054	10.057	-5.757
C	-0.735	2.364	-0.217
C	-0.501	3.481	-10.02
H	-0.081	3.22	-0.436
C	4.105	2.514	8.446
C	-4.158	8.518	-7.906
C	-0.437	3.619	-8.523
C	5.850	4.586	0.079
H	3.864	3.898	0.533
C	3.934	-1.385	-1.954
H	-1.753	3.55	2.927
N	6.654	-4.17	5.355
H	5.062	1.897	-0.278
H	5.095	-4.194	2.669
H	4.009	4.66	-1.045
H	2.731	2.73	-2.050
H	5.005	1.897	-3.044
H	7.413	2.716	-1.342
H	7.754	3.139	-3.795
H	-0.266	3.189	-3.63
H	-0.657	0.345	-4.536
H	-0.08	1.565	0.163
H	-2.386	3.559	0.502
H	5.559	-2.399	4.109
C	-0.523	1.300	-2.597
C	-1.439	1.877	-1.494
H	0.138	0.554	-2.131
H	-2.039	2.697	-1.918
H	-2.158	1.101	-1.206
N	-5.161	9.175	-11.499
C	6.360	-2.401	8.232
N	6.671	-2.624	9.333

4. Fitting parameters in Figure 5

Table S6. Fitting parameters of the TRPL decay shown in Figure 5a.

Excitation Fluence (photons/(pluse×cm^2))	Amplitude A_1	Lifetime τ_1 (ps)
4.8×10^{12}	1.053	822 ± 17
1.0×10^{13}	1.084	794 ± 18
1.6×10^{13}	1.081	884 ± 16

Table S7. Fitting parameters of the TA decay shown in Figure 5b.

Excitation Fluence (photons/(pluse×cm^2))	Amplitude A_1	Lifetime τ_1 (ps)	Amplitude A_2	Lifetime τ_2 (ps)	Amplitude A_3	Lifetime τ_3 (ps)
2×10^{14}	4.7	1.6 ± 0.2	2.8	31 ± 2	0.58	3929 ± 1155

References

1. Stephens, P.J.; Devlin, F.J.; Chabalowski, C.F.; Frisch, M.J. Ab initio calculation of vibrational absorption and circular dichroism spectra using density functional force fields. *J. Phys. Chem.* **1994**, *98*, 11623–11627, doi:10.1021/j100096a001.
2. Rassolov, V.A.; Pople, J.A.; Ratner, M.A.; Windus, T.L. 6-31G* basis set for atoms K through Zn. *J. Chem. Phys.* **1998**, *109*, 1223–1229, doi:10.1063/1.476673.
3. Frisch, M.J.; Trucks, G.W.; Schlegel, H.B.; Scuseria, G.E.; Robb, M.A.; Cheeseman, J.R.; Scalmani, G.; Barone, V.; Petersson, G.A.; Nakatsuji, H., et al. *Gaussian Inc.* **2016**, Wallingford, CT. https://gaussian.com/g09citation/ (accessed on 1 November,2016)
4. Weigend, F.; Ahlrichs, R. Balanced basis sets of split valence, triple zeta valence and quadruple zeta valence quality for H to Rn: Design and assessment of accuracy. *Phys. Chem. Chem. Phys.* **2005**, *7*, 3297–3305, doi:10.1039/b508541a.
5. Martin, R.L. Natural transition orbitals. *J. Chem. Phys.* **2003**, *118*, 4775–4777, doi:10.1063/1.1558471.
6. Lu, T.; Chen, F. Multiwfn: A multifunctional wavefunction analyzer. *J. Comput. Chem.* **2012**, *33*, 580–592, doi:10.1002/jcc.22885.
7. Humphrey, W.; Dalke, A.; Schulten, K. VMD: visual molecular dynamics. *J. Molec. Graphics* **1996**, *14*, 33–38, doi:10.1016/0263-7855(96)00018-5.
8. Liu, Z.; Lu, T.; Chen, Q. An sp-hybridized all-carboatomic ring, cyclo[18]carbon: Electronic structure, electronic spectrum, and optical nonlinearity. *Carbon* **2020**, *165*, 461–467, doi:10.1016/j.carbon.2020.05.023.
